# Genome-Wide Association Study of Relative Telomere Length

**DOI:** 10.1371/journal.pone.0019635

**Published:** 2011-05-10

**Authors:** Jennifer Prescott, Peter Kraft, Daniel I. Chasman, Sharon A. Savage, Lisa Mirabello, Sonja I. Berndt, Joel L. Weissfeld, Jiali Han, Richard B. Hayes, Stephen J. Chanock, David J. Hunter, Immaculata De Vivo

**Affiliations:** 1 Channing Laboratory, Department of Medicine, Brigham and Women's Hospital, Harvard Medical School, Boston, Massachusetts, United States of America; 2 Program in Molecular and Genetic Epidemiology, Department of Epidemiology, Harvard School of Public Health, Boston, Massachusetts, United States of America; 3 Donald W. Reynolds Center for Cardiovascular Research, Brigham and Women's Hospital, Harvard Medical School, Boston, Massachusetts, United States of America; 4 Division of Cancer Epidemiology and Genetics, Department of Health and Human Services, National Cancer Institute, National Institutes of Health, Bethesda, Maryland, United States of America; 5 University of Pittsburgh Cancer Institute, Pittsburgh, Pennsylvania, United States of America; 6 Department of Dermatology, Brigham and Women's Hospital, Harvard Medical School, Boston, Massachusetts, United States of America; 7 Division of Epidemiology, New York University Medical Center, New York, New York, United States of America; 8 Program in Medical and Population Genetics, Broad Institute of Harvard and MIT, Cambridge, Massachusetts, United States of America; Universite de Montreal, Canada

## Abstract

Telomere function is essential to maintaining the physical integrity of linear chromosomes and healthy human aging. The probability of forming proper telomere structures depends on the length of the telomeric DNA tract. We attempted to identify common genetic variants associated with log relative telomere length using genome-wide genotyping data on 3,554 individuals from the Nurses' Health Study and the Prostate, Lung, Colorectal, and Ovarian Cancer Screening Trial that took part in the National Cancer Institute Cancer Genetic Markers of Susceptibility initiative for breast and prostate cancer. After genotyping 64 independent SNPs selected for replication in additional Nurses' Health Study and Women's Genome Health Study participants, we did not identify genome-wide significant loci; however, we replicated the inverse association of log relative telomere length with the minor allele variant [C] of rs16847897 at the *TERC* locus (per allele β = −0.03, P = 0.003) identified by a previous genome-wide association study. We did not find evidence for an association with variants at the *OBFC1* locus or other loci reported to be associated with telomere length. With this sample size we had >80% power to detect β estimates as small as ±0.10 for SNPs with minor allele frequencies of ≥0.15 at genome-wide significance. However, power is greatly reduced for β estimates smaller than ±0.10, such as those for variants at the *TERC* locus. In general, common genetic variants associated with telomere length homeostasis have been difficult to detect. Potential biological and technical issues are discussed.

## Introduction

Two major challenges are encountered in maintaining the physical integrity of linear chromosomes. First, cells must prevent the recognition of chromosome ends as double-stranded DNA breaks [1]. Second, telomeric ends are lost with each cell division as a result of the end replication problem [2]. On average, human telomeres lose 50 to 100 bp per mitotic division, which limits the replicative capacity of a cell [3]. To overcome these issues, eukaryotic cells evolved the telomere maintenance system, the almost universal mechanism used to protect chromosome ends [4].

Telomeres are complex dynamic nucleoprotein structures that consist of a long stretch of hexameric (TTAGGG)_n_ DNA repeats, a single-stranded G-rich 3′ overhang, the telomerase enzyme complex, six telomere-specific proteins, and other telomere-associated proteins including those of the DNA repair machinery [5]. In normal mitotic cells, telomeres switch between capped and uncapped states [6]. Telomere-associated proteins assist the single-stranded G-rich 3′ overhang in invading the double-stranded telomeric tract to form a large terminal loop. This conformation contributes to the functional capped structure of the telomeric end, inhibiting the DNA damage response [7]. Telomerase, a highly conserved enzyme consisting of protein [telomerase reverse transcriptase (*TERT*), dyskerin (*DKC1*)] and RNA [telomerase RNA component (*TERC*)] components [8], appends chromosome ends with hexameric repeats to restore telomere length [9] and plays a role in stabilizing telomeres in the capped state [6].

The likelihood of a telomere existing in an uncapped vs. a capped state is dependent on telomere length. Longer telomeres are more likely to form the protective capped structure than shorter telomeres [6]. In germ cells, strong telomerase expression maintains long telomere lengths [10]. Telomerase activity has been detected at lower levels in fibroblasts, peripheral blood leukocytes, skin, hair follicles, intestinal crypts, and endometrium. In these tissues, expression levels are not sufficient to prevent replication-associated telomere attrition [11]–[15]. As a result, telomere length declines with age [16]–[21]. As telomeres shorten and become dysfunctional, the uncapped chromosomal ends undergo nucleolytic decay, chromosomal end-to-end fusions, and atypical recombination triggering senescence or apoptosis [22].

Diseases characterized by telomere dysfunction highlight the importance of telomere maintenance for healthy human aging. Patients with dyskeratosis congenita, a rare inherited bone marrow failure and cancer predisposition syndrome, have very short germline telomeres (<1^st^ percentile for age), and approximately one-half have a mutation in a known telomere biology gene [23]. Patients with the shortest telomeres exhibit the most severe phenotypes [24]. Telomere attrition, genomic instability, and premature senescence are also features of Hutchinson-Gilford progeria syndrome and Werner syndrome [25]. Shorter leukocyte telomere length has been implicated in a number of common aging-related diseases such as cancer [26]–[30] and cardiovascular disease [31]–[36] as well as increased mortality [37]–[41].

Despite the essential role telomeres play in the maintenance of normal cellular function, little is known about the common genetic determinants of telomere length. Heritability estimates from family and twin studies for telomere length range from 36% to 86% [17], [40]–[45]. Telomere lengths in different tissues are significantly correlated, with far less variation between tissues from the same individual compared to variation between individuals of a particular tissue type [18], [20], [21], [46]–[48]. Quantitative trait linkage analyses identified significant linkage to chromosomes 12p11.2 [43], [49], and 14q23.2 [44], but these loci have not been replicated in independent studies [44], [50]–[52]. Common genetic variants in known telomere maintenance genes do not exhibit strong influences on telomere length [45], [53]–[55]. Genome-wide association studies (GWAS) have identified and replicated associations at the *TERC* locus, which account for no more than 1% of variation in telomere length [51], [52]. Variants at the oligonucleotide/oligosaccharide-binding fold containing 1 (*OBFC1*) gene locus, which codes for a protein that interacts with telomere-specific proteins and is implicated in telomere length regulation [56], were also recently identified using a meta-analysis of GWAS cohorts [52]. To identify additional variants associated with telomere length, we conducted a GWAS using data from two large cohorts that took part in the National Cancer Institute Cancer Genetic Markers of Susceptibility (CGEMS) initiative for breast and prostate cancer.

## Results

**Figure 1:**
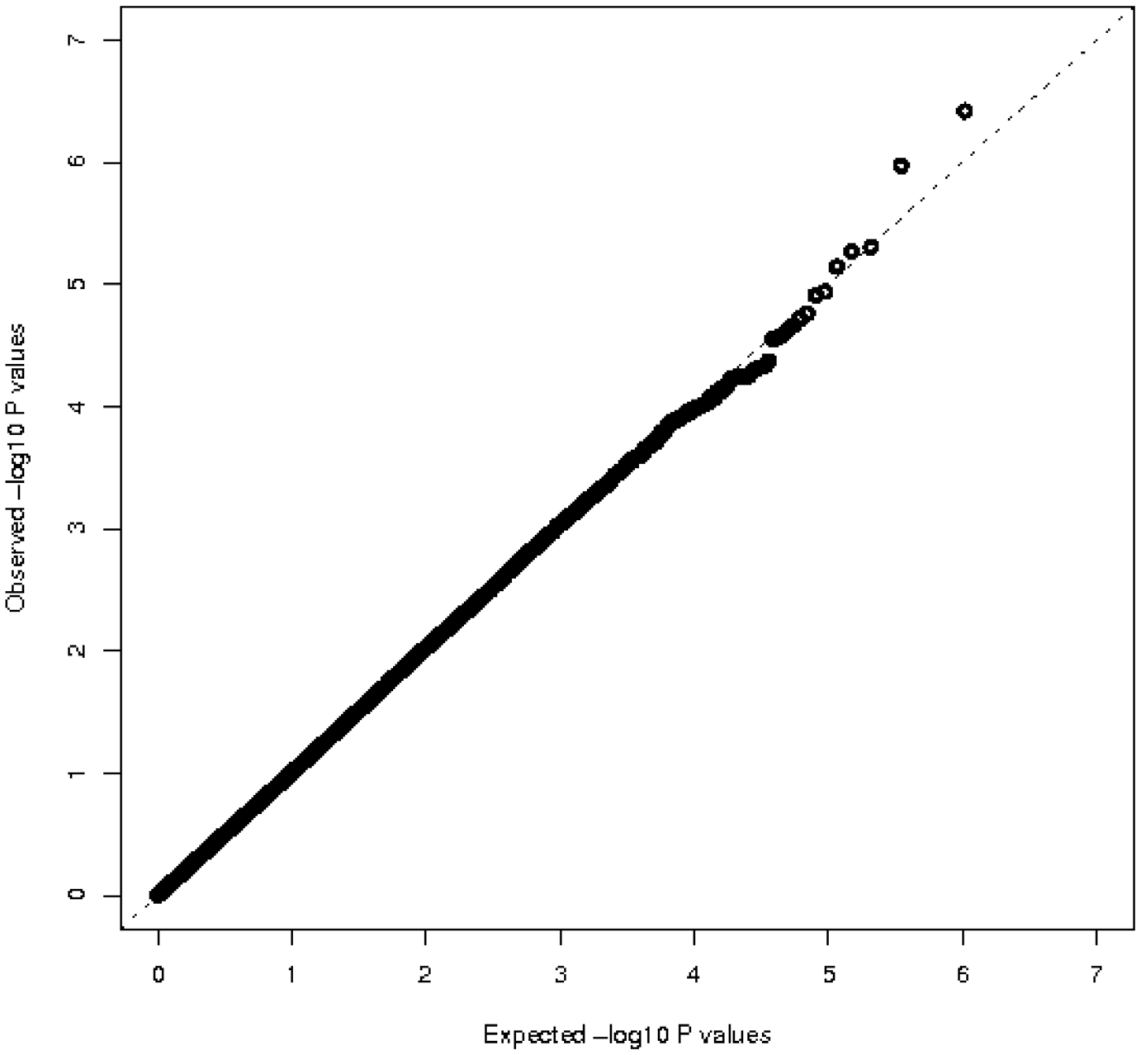
Log Quantile-Quantile (Q-Q) P value plot. The observed –log10 P values (Y-axis) of 519,076 SNPs from the pooled NHS and PLCO GWAS data set adjusted for the principal components of genetic variation plotted against the expected –log10 quantile (X-axis). The dashed line represents imputed P values.

We used a two-stage GWAS to identify common genetic variants associated with log relative telomere length. Age, smoking, and mean log relative telomere length values of each population are shown in **Table 1**. The GWAS discovery set included genotypes for 519,076 SNPs on a total of 3554 individuals from the Nurses' Health Study (NHS) and the Prostate, Lung, Colorectal, and Ovarian (PLCO) Cancer Screening Trial. After adjusting for the top principal components of genetic variation from each cohort, the distribution of the observed P values does not suggest any inflation in Type 1 error rates due to population stratification or other sources of bias (**Figure 1**).

**Table 1:** Characteristics of GWAS and replication populations.

		NHS GWAS	PLCO GWAS	NHS Replication	WHS Replication
No. subjects		1944	1610	1345	1115
Mean age (range)		58.5 (43–70)	63.9 (55–74)	58.4 (43–69)	56.0 (45–82)
Smoking status (%)					
	Never	46.2	39.9	46.2	49.7
	Former	40.5	50.1	41.9	36.7
	Current	13.3	9.9	12.0	13.6
Pack-years (%)*					
	1–9	32.3	21.1	33.8	43.8
	10–29	34.9	36.5	34.9	39.8
	30+	32.7	42.4	31.2	16.4
Mean log(RTL) (SD)					
	Cases	2.78 (0.35)	2.59 (0.55)	2.31 (0.35)	2.32 (0.26)
	Controls	2.79 (0.35)	2.58 (0.58)	2.30 (0.34)	2.31 (0.25)

*among participants who have ever smoked.

None of the P values observed in the first stage of our GWAS reached the genome-wide significance level of P<5.0×10^−8^ [57]. The SNP most significantly associated with log relative telomere length in this discovery set was rs1978423, located upstream of the pyroglutamyl-peptidase I (*PGPEP1*) and LSM4 homolog, U6 small nuclear RNA associated (*LSM4*) genes on chromosome 19 (P = 3.8×10^−7^). We selected the 64 independent SNPs most significantly associated with log relative telomere length to genotype in additional samples in the NHS (N = 1345) and the Women's Genome Health Study (WGHS) (N = 1115) replication populations. Estimates for all SNPs exhibited moderate to high between-study heterogeneity (I^2^≥0.50; **Table S1**). rs1978423 was not associated with log relative telomere length in either replication population (P≥0.12). Two SNPs, rs4073054 at the alipoprotein A-II (*APOA2*)/nuclear receptor subfamily 1, group I, member 3 (*NR1I3*) locus on chromosome 1 and rs975504 near dehydrogenase/reductase (SDR family) member 12 (*DHRS12*) and a predicted non-coding RNA on chromosome 13, displayed estimates similar to the associations from the initial GWAS population with P values <0.05 in the NHS replication population, but not within the WGHS (**Table S1**; joint P values of GWAS and replication populations are 1.7×10^−4^ and 9.5×10^−4^, respectively). rs7210405, ∼24 kb downstream of the cytohesin 1 (*CYTH1*) gene on chromosome 17, displayed the smallest P value (0.01) within the WGHS population with a similar estimate. However, this SNP was not associated with log relative telomere length in the NHS replication population (joint P = 1.4×10^−4^).

We examined SNPs at the *TERC* and *OBFC1* loci, which were recently identified by published GWAS of telomere length [51], [52]. Within our discovery GWAS and WGHS replication populations, we examined associations with genotyped and imputed SNPs (rs12696304, rs10936599, rs3772190, rs16847897) at the *TERC* locus reported as significantly associated with telomere length in prior GWAS. In our study populations, we found evidence for associations between log relative telomere length and SNPs at this locus, with the most significant association observed for the minor allele [C] of rs16847897 (per allele β = −0.03, joint P = 0.003). Meta-analysis of our results for rs16847897 compared with study-specific estimates from published GWAS generated a P = 1.6×10^−13^ and displayed virtually no between-study heterogeneity (**Table 2**). Whereas Levy et al. [52] reported genome-wide significant associations with SNPs in the *OBFC1* gene, we did not find evidence for associations between genotyped or imputed SNPs at the *OBFC1* locus and log relative telomere length in our study (joint P≥0.12). Meta-analysis of our results for SNPs at the *OBFC1* locus with those reported by Levy et al. demonstrated high between-study variability (**Table 2**). As we previously reported, we did not find associations between log relative telomere length and common genetic variants at the *TERT-CLPTM1L*, *BICD1*, and *DDX11* loci [55].

**Table 2:** Relative telomere length associations with SNPs at loci identified by published GWAS.

			CGEMS GWAS*			WGHS replication†			Meta-analysis‡				
Locus	SNP	WT/VT	MAF	β (s.e.)	P value	MAF	β (s.e.)	P value	β (s.e.)	P value	Q	I^2^	P_heterogeneity_
TERC	rs12696304	C/G	0.27	−0.001 (0.01)	9.1E-01	0.26	−0.03 (0.01)	3.4E-02	−**0.03 (0.004)**	**1.5E-14**	**15.35**	**0.54**	**3.2E-02**
TERC	rs10936599	C/T	0.25	−0.02 (0.01)	1.9E-01	0.24	−0.03 (0.01)	3.2E-02	−**0.03 (0.008)**	**2.8E-04**	**5.39**	**0.63**	**6.7E-02**
TERC	rs3772190	G/A	0.25	−0.02 (0.01)	1.9E-01	0.24	−0.03 (0.01)	3.8E-02	−**0.03 (0.008)**	**3.3E-04**	**5.40**	**0.63**	**6.7E-02**
TERC	rs16847897	G/C	0.28	−0.03 (0.01)	2.1E-03	0.28	−0.01 (0.01)	2.7E-01	−**0.03 (0.004)**	**1.6E-13**	**5.83**	**0.00**	**4.4E-01**
OBFC1	rs11191839	G/A	0.35	−0.001 (0.01)	9.2E-01	0.36	−0.006 (0.01)	6.0E-01	−0.003 (0.008)	0.66	0.10	0.00	7.5E-01
OBFC1	rs2902638	T/C	0.25	0.002 (0.01)	8.6E-01	0.25	−0.03 (0.01)	2.6E-02	−0.01 (0.009)	0.16	3.03	0.67	8.2E-02
OBFC1	rs10748858	T/G	0.41	−0.004 (0.01)	7.1E-01	0.41	0.006 (0.01)	6.1E-01	0.0005 (0.008)	0.95	0.40	0.00	5.3E-01
OBFC1	rs2067832	T/C	0.50	−0.002 (0.01)	8.4E-01	N/A							
OBFC1	rs9325507	T/C	0.50	−0.002 (0.01)	8.7E-01	0.50	0.006 (0.01)	6.1E-01	**0.02 (0.007)**	**1.1E-02**	**19.45**	**0.74**	**1.6E-03**
OBFC1	rs1980653	G/A	0.50	−0.003 (0.01)	8.1E-01	0.50	0.006 (0.01)	6.1E-01	**0.008 (0.007)**	**2.3E-01**	**25.17**	**0.80**	**1.0E-04**
OBFC1	rs4918069	T/G	0.27	0.004 (0.01)	7.4E-01	0.29	−0.03 (0.01)	4.3E-02	−0.01 (0.008)	0.26	2.92	0.66	8.8E-02
OBFC1	rs2984132	T/C	0.40	−0.002 (0.01)	8.5E-01	0.40	−0.009 (0.01)	4.2E-01	−0.005 (0.008)	0.49	0.21	0.00	6.5E-01
OBFC1	rs2487999	C/T	0.10	0.002 (0.02)	9.2E-01	0.10	0.04 (0.02)	3.4E-02	**0.04 (0.01)**	**1.1E-04**	**19.94**	**0.75**	**1.3E-03**
OBFC1	rs3752949	A/G	0.23	−0.0006 (0.01)	9.6E-01	0.22	0.03 (0.01)	4.4E-02	0.01 (0.009)	0.18	2.24	0.55	1.3E-01
OBFC1	rs11191865	G/A	0.50	0.001 (0.01)	9.1E-01	0.49	−0.006 (0.01)	6.1E-01	−0.002 (0.008)	0.80	0.21	0.00	6.5E-01
OBFC1	rs1265164	G/A	0.14	−0.008 (0.02)	6.0E-01	0.07	0.02 (0.02)	4.3E-01	−0.0003 (0.01)	0.98	0.88	0.00	3.5E-01
OBFC1	rs9419958	C/T	0.14	−0.006 (0.02)	7.1E-01	0.14	0.03 (0.02)	1.0E-01	**0.03 (0.01)**	**5.3E-04**	**23.72**	**0.79**	**2.0E-04**
OBFC1	rs9420907	A/C	0.14	−0.004 (0.02)	7.8E-01	0.14	0.03 (0.02)	1.0E-01	**0.03 (0.01)**	**4.3E-04**	**23.27**	**0.79**	**3.0E-04**
OBFC1	rs4387287	C/A	0.15	−0.005 (0.02)	7.3E-01	0.14	0.03 (0.02)	6.3E-02	**0.04 (0.01)**	**1.1E-05**	**26.55**	**0.77**	**2.0E-04**
OBFC1	rs17813699	G/A	0.10	−0.005 (0.02)	7.5E-01	0.12	−0.02 (0.02)	4.5E-01	−0.01 (0.01)	0.48	0.18	0.00	6.7E-01
OBFC1	rs1342214	C/T	0.40	0.003 (0.01)	7.9E-01	0.41	−0.007 (0.01)	5.2E-01	−0.002 (0.008)	0.79	0.42	0.00	5.2E-01

*β estimates and P values derived from linear regression adjusted for age at blood collection, gender, an age-gender interaction term, smoking status, pack-year categories of smoking, disease status, and principal components of genetic variation.

† β estimates and P values derived from linear regression adjusted for age at blood collection, smoking status, pack-year categories of smoking, disease status, and principal components of genetic variation; N/A  =  SNP could not be imputed.

‡ Combined effect sizes and P values are calculated using a fixed-effect meta-analysis. Estimates in bold are based upon meta-analysis of the current study populations along with those published by Codd et al. and Levy et al. Study-specific values from the published studies were used where available.

## Discussion

Despite the high heritability of telomere length [17], [40]–[45], common variants associated with large effects have remained elusive. We were unable to identify novel variants associated with log relative telomere length with a high degree of confidence among a total of 6,014 participants. The two most promising SNPs identified by our study were rs4073054 (NHS GWAS, β = −0.04, P = 1.6×10^−4^; PLCO GWAS, β = −0.04, P = 0.041; NHS replication, β = −0.03, P = 0.01) and rs7210405 (NHS GWAS, β = 0.03, P = 0.006; PLCO GWAS, β = 0.05, P = 0.005; WGHS replication, β = 0.03, P = 0.01), as 3 of our 4 study populations displayed evidence of an association with log relative telomere length. We had >80% power to detect β estimates as small as ±0.10 for SNPs with minor allele frequencies (MAF) of ≥0.15 in our discovery GWAS population (N = 3554). However, published associations with relative telomere length suggest effect estimates may be quite a bit smaller than ±0.10. Our study was underpowered to detect genome-wide significant associations between log relative telomere length and SNPs of much weaker effect. For example, adequate power to detect genome-wide significant associations between SNPs at loci such as *TERC* that have a MAF ∼0.25, β∼0.03, and log relative telomere length as measured in our study would require a sample size of ∼25,000 unrelated individuals. Even to reach the suggestive threshold of α = 1×10^−5^, to detect an association with such a SNP would require a sample size of 18,000 individuals, roughly 5 x greater than the size of our initial GWAS population. Discovery of additional loci with small effect sizes will be feasible once meta-analyses of existing and future GWAS of telomere length are conducted.

To date, only the *TERC* locus has been identified and replicated in multiple independent populations for its association with telomere length [51], [52]. Although SNPs within the *TERC* locus did not reach genome-wide significance in the discovery GWAS population (N = 2917) of Codd et al. [51], rs16847897 fell marginally outside of their pragmatic significance threshold (P<1×10^−5^) for replication. Due to the biological significance of the locus, the authors examined and confirmed associations between rs16847897 and a second SNP at the locus, rs12696304, with telomere length in 3 replication populations. Levy et al. found additional evidence for associations between SNPs at the *TERC* locus and telomere length [52]. We found evidence for associations with SNPs at the *TERC* locus, observing the strongest association between the minor allele [C] of rs16847897 and log relative telomere length (joint P = 0.003) with an effect size and direction consistent with that of Codd et al. Similar to Codd et al., we did not find associations between log relative telomere length and SNPs at loci previously reported to be associated with telomere length [43], [49], [50], [58], including the *OBFC1* locus recently identified by a similarly sized GWAS of telomere length [52].

If yeast genetic mapping studies are any indication [59], [60], hundreds of genes regulate human telomere length homeostasis. Given that known telomere maintenance genes are highly conserved [61] and mutations have deleterious consequences [23], most functional common variants (> 5%) will likely have small effects. Additionally, recent studies demonstrate that while telomere length of offspring is significantly correlated with maternal [62], [63] and paternal [41], [64], [65] telomere lengths, the paternal contribution appears to be much stronger [41], [64], [65] and positive correlations have been observed between paternal age at birth and offspring telomere length [41], [66]–[68]. An influence of imprinted genes in regulating telomere length could account for these observations. By not controlling for parental origin of alleles at imprinted loci, SNP effects, which are likely to be small to begin with, would be underestimated and the power to detect associations reduced [69]. For many GWAS populations, including ours, determining the parental origin of alleles is not feasible.

Evidence suggests cis-acting factors in subtelomeric regions regulate telomere length. While chromosome-specific telomeres tend to be similar between different individuals [48], [70]–[73], allele specific lengths are inherited [74] and may differ by more than 6 kb at the same telomere [75]. A substantial amount of genetic diversity is provided by subtelomeric regions, which are riddled with repetitive DNA, segmental duplications, and large copy number polymorphisms including variable copy numbers of functional genes. Knowledge of subterminal sequences is still incomplete and the complexity within these regions has complicated sequencing efforts [76] resulting in low coverage of such regions on commercially available genome-wide genotyping arrays [77].

Technical challenges further complicate the search for genetic variants of telomere length. While the quantitative PCR-based assay is the most economical, high-throughput method for telomere length measurements in large epidemiologic studies [78], values are relative representations of the average telomere length. The telomere restriction fragment (TRF) assay provides absolute values for the average, shortest, and longest telomere lengths. However, TRF measurements include variable amounts of the subtelomeric region, are time and labor intensive, and require much more genomic DNA than the PCR-based assay [79]. The single telomere length analysis (STELA) assay is currently the most sensitive assay, developed with the potential to provide allele-specific telomere lengths [75]. To date, telomere and allele-specific STELA assays have only been developed for a very small fraction of chromosome ends due to incomplete knowledge of subtelomeric regions [73].

The inability to capture the complexity of telomere length regulation with a single measurement is a major obstacle to GWAS efforts. Even the well established decline in telomere length with increasing age is not a simple linear relationship. Relatively fast rates of telomere attrition are observed during childhood and adolescence. Attrition rates slow creating a plateau from ∼30 to 50 years of age followed by another decline in telomere length from about age 50 to 80 [19], [64], [80], [81]. It is unknown whether temporally regulated genes and/or environmental exposures are responsible for the change in attrition rates. The age range of individuals in our GWAS population (**Table 1**) and those of previously published studies [50]–[52] span these different age periods potentially diluting telomere length associations with variants that influence temporally regulated genes.

Oxidative stress is thought to accelerate telomere attrition as a result of damage to telomeric DNA, which is less efficiently repaired [82]. Systemic exposures that contribute to oxidative stress such as smoking [62], [83], [84] and obesity [54], [84]–[87] have been associated with shorter telomeres. Whereas, healthy lifestyle choices promote a more stable telomere length [81], [86], [88], possibly by increasing telomerase activity in cells [89]. Evidence suggests environmental factors may take on a more prominent role as determinants of telomere length with increasing age [65], [90], but the relationship with these exposures are not yet clearly defined as many studies fail to find significant correlations between telomere length and smoking [33], [40], [44], [54], [91], obesity [92], [93], and/or physical activity [92], [94]. Since pack-years of smoking were significantly correlated with relative telomere length among men of the PLCO GWAS population, we adjusted for smoking characteristics in all of our regression models. Although relative telomere length in our prospectively collected blood samples was not associated with breast [93] or prostate cancer [88], we adjusted for case status to control for any potential effects of pre-clinical disease on telomere length.

In summary, improved sequence maps and technical capabilities are necessary to increase success in identifying and validating common genetic variants associated with telomere length homeostasis. Efforts will likely require meta-analyses of existing and future telomere length GWAS to increase power to detect common variants of small effects while stratifying by age groups defined by attrition rate and controlling for environmental factors.

## Materials and Methods

### Ethics Statement

The NHS and WGHS study protocols were approved by the Committee on Use of Human Subjects of the Brigham and Women's Hospital, Boston, MA. Institutional review boards at the U.S. National Cancer Institute and the 10 screening centers approved the PLCO protocol.

### NHS breast cancer GWAS population

The NHS is a prospective cohort study of 121,700 female registered nurses in 11 states in the United States who were 30–55 years of age at enrollment. In 1976 and biennially thereafter, detailed information from participants was collected by self-administered questionnaires. Participants in this study were selected for a nested case-control study of telomere length and postmenopausal breast cancer risk from the subcohort of 32,826 women who donated a blood sample in 1989–90 [93]. Eligible cases consisted of postmenopausal women of European ancestry with pathologically confirmed incident invasive breast cancer diagnosed anytime after blood collection up to June 1, 2004 with no prior diagnosis of cancer. Controls were randomly selected postmenopausal women free of cancer and matched to cases according to age, blood collection, and ethnicity. Completion of the questionnaire and submission of the blood sample was considered to imply informed consent. No significant difference was observed for log relative telomere length between 1,122 cases and 1,147 controls and no significant association with postmenopausal breast cancer risk [93]; therefore, we included telomere length data from both breast cancer cases and controls for the current study.

### PLCO prostate cancer GWAS population

The PLCO Cancer Screening Trial is an ongoing randomized trial with 154,942 persons aged 55 to 74 enrolled between September 1993 and July 2001 from 10 screening centers nationwide [95]. Detailed questionnaire data was collected from all subjects at baseline. Participants provided blood and tissue samples for etiologic studies of cancer [96] and all participants provided written informed consent. Participants in this study were male subjects selected for a nested case-control study of telomere length, prostate cancer risk, and life-style variables [88]. Eligible cases and controls consisted of non-Hispanic white men aged 55 to 74 who had been screened for prostate cancer (PSA test) prior to October 1, 2003, completed a baseline questionnaire of cancer risk factors, provided a blood sample 1 month to 3 years prior to prostate cancer diagnosis for cases, and did not have a personal history of cancer prior to study entry. All cases had pathologically confirmed incident aggressive prostate cancer and a Gleason score of ≥7. No significant difference was observed for relative telomere length between 616 cases and 1,061 matched controls and no significant association with aggressive prostate cancer risk [88]; therefore, we included telomere length data from both prostate cancer cases and controls for the current study.

### Replication populations

NHS participants in the replication phase of this study were selected for a nested case-control study of telomere length and skin cancer risk from the blood subcohort [97]. Eligible cases were women of European ancestry with skin cancer diagnosed anytime after blood collection up to June 1, 2000 with no prior diagnosis of skin cancer. A common control series was randomly selected from participants who gave a blood sample and were free of diagnosed skin cancer up to and including the questionnaire cycle in which the case was diagnosed. Controls were matched to cases by age and ethnicity. The nested case-control study consisted of 218 melanoma cases, 285 cases with squamous cell carcinoma, 300 cases with basal cell carcinoma, and 870 matched controls. The study protocol was approved by the Committee on Use of Human Subjects of the Brigham and Women's Hospital, Boston, MA.

The Women's Genome Health Study (WGHS) is a prospective cohort of female North American health care professionals representing participants in the Women's Health Study (WHS) who provided a blood sample at baseline and consent for blood-based analyses [98]. The WHS was a 2×2 trial beginning in 1992–1994 of vitamin E and low dose aspirin in prevention of cancer and cardiovascular disease with about 10 years of follow-up. Follow-up continues in observational mode. Participants in the WHS were 45 or older at enrollment and free of cardiovascular disease, cancer or other major chronic illness and were followed prospectively for the influence of random allocation of vitamin E on cancer [99]–[101]. Additional information related to health and lifestyle were collected by questionnaire throughout the WHS trial and continuing observational follow-up. Participants in the current analysis are individuals selected for a breast cancer case-control study nested within the WGHS. Eligible cases consisted of women of European ancestry diagnosed with pathologically confirmed incident invasive breast cancer until March 7, 2000. Controls were randomly selected participants who had given a blood sample, and were free of diagnosed cancer. Controls were matched to cases according to age, menopausal status, postmenopausal hormone use at time of blood draw, and race/ethnicity. Written informed consent was obtained from all women before their entry into the trial.

### Genotyping and quality control

As part of the CGEMS initiative, genotyping of the NHS samples was conducted for the first stage of a three-stage GWAS of breast cancer susceptibility using the Illumina HumanHap550 Infinium assay (Illumina, San Diego, CA), which contains SNPs derived from the HapMap phase I and II data [102]. Genotyping of PLCO samples for the prostate cancer susceptibility project occurred in two parts [103]. Phase 1A used Illumina's Sentrix HumanHap300 assay and Phase 1B used the Sentrix HumanHap240 assay. For each cohort, samples with call rates <90% and single nucleotide polymorphism (SNP) assays with call rates under 90% were removed. Polymorphisms with a minor allele frequency of <1% were removed.

Genotyping of WGHS samples was performed using the Illumina HumanHap300 Duo ‘‘+’’ chips or the combination of the HumanHap300 Duo and iSelect chips. In either case, the custom SNP content was the same; these custom SNPs were chosen without regard to minor allele frequency (MAF) to saturate candidate genes for cardiovascular disease as well as to increase coverage of SNPs with known or suspected biological function, e.g. disease association, non-synonymous changes, substitutions at splice sites, etc. For quality control, all samples were required to have successful genotyping using Illumina's BeadStudio v. 3.3 software for at least 98% of the SNPs. Self-reported European ancestry was verified on the basis of multidimensional scaling analysis of identity by state using 1443 ancestry informative markers in PLINK v. 1.06. The final data set retained SNPs with MAF >1%, successful genotyping in 90% of the subjects, and deviations from Hardy-Weinberg equilibrium not exceeding P = 10^−6^ in significance. Among the final 23,294 individuals of verified European ancestry, genotypes for a total of 2,608,509 SNPs were imputed from the experimental genotypes and LD relationships implicit in the HapMap r. 22 CEU samples.

Genotyping of SNPs selected for replication in the NHS nested skin cancer case-control data set was performed at the Dana Farber/Harvard Cancer Center High-Throughput Genotyping Core. Whole genome amplified DNA was genotyped using the Taqman® OpenArray® Real-Time qPCR system (Applied Biosystems Inc, Foster City, CA). Of the 64 SNPs selected for replication, one failed to genotype. We removed 1 SNP with a call rate <90%. All SNPs were tested for deviation from Hardy-Weinberg equilibrium within the entire skin cancer case-control data set. We observed significant deviations for 3 SNPs at the Bonferroni-adjusted P<0.0008, which were excluded from analyses. We included 5% blinded quality control samples to validate genotyping procedures; concordance for blinded samples was >99%.

### Relative telomere length measurement

Genomic DNA was extracted from peripheral blood leukocytes using the QIAmp (Qiagen, Chatsworth, CA) 96-spin blood protocol. DNA was quantified using either the Molecular Devices 96-well spectrophotometer (NHS, WGHS) or the Nanodrop SD-1000 spectrophotometer (PLCO), and subsequently dried down and resuspended to ensure accurate and uniform DNA concentrations. Relative telomere length was measured using a previously described modified version [27] of the quantitative PCR-based telomere assay [78]. Briefly, 5 ng of genomic DNA was dried down in a 384-well plate and resuspended in 10 µL of either the telomere (T) or 36B4 (S; single copy gene) PCR reaction mixture. The Telomere reaction mixture consists of 1x QuantiTect® SYBR® Green PCR Master Mix (Qiagen), 2 mM of DTT, 270 nM Tel-1b primer, and 900 nM Tel-2b primer. The Telomere thermal cycling profile proceeds as follows: 95°C for 10 minutes then 30 cycles consisting of 95°C for 15 seconds and 54°C for 2 minutes. The 36B4 reaction mixture consists of 1x QuantiTect® SYBR® Green PCR Master Mix, 300 nM 36B4u primer, and 500 nM 36B4d primer. The 36B4 thermal cycling profile proceeds as follows: 95°C for 10 minutes then 30 cycles consisting of 95°C for 15 seconds and 58°C for 1 minute and 10 seconds. The threshold cycle (Ct) value for each reaction represents the number of PCR cycles required to detect a signal over background fluorescence and is inversely proportional to the amount of starting DNA. Assuming 100% PCR efficiency, the amount of PCR product exactly doubles with each cycle. Triplicate reactions of each assay were performed on each sample and the average of the 3 measurements was used for analyses. Relative telomere length is calculated as the exponentiated ratio of the telomere repeat copy number to single-gene copy number (2^-T/S^) and represents the average telomeric DNA signal per genome of an individual. Coefficients of variation (CV) for the telomere and single-gene assay ranged from 0.66% to 3.02% and 0.56% to 2.07%, respectively.

### Statistical analyses

Continuous relative telomere length was natural logarithm transformed to satisfy the assumption of normality. We used multivariable linear regression to analyze the additive effect of each SNP (0, 1, or 2 copies of minor allele) on log relative telomere length using the pooled NHS and PLCO GWAS data set. As log relative telomere length is inversely associated with age in all populations within this study [88], [93], [97] and significantly inversely associated with smoking in the PLCO population, which included a higher proportion of heavier smokers (**Table 1**) [88], the regression model included age as a continuous variable, smoking status, and pack-year categories of smoking (never, <10, 10 to <30, 30+) in addition to disease status. Since most published studies find shorter telomere lengths among adult men compared to women [42], [43], [62], [65], [92], [104], [105] as well as potentially faster rates of telomere attrition among men [66], [67], [92], we included gender and an age-gender interaction term in the initial GWAS linear regression analyses. To control for potential confounding by population stratification in the NHS GWAS, PLCO GWAS, and WGHS replication data sets, we additionally adjusted for the top principal components of genetic variation chosen for each study after excluding any admixed individuals clearly not of European descent. Principal components of genetic variation were calculated with EIGENSTRAT software [106] as described in Hunter et al, 2007 [102]. All P values are two-sided. Statistical analyses were performed with SAS (version 9.1; SAS Institute, Cary, NC) and PLINK [107]. Power calculations were performed using QUANTO [108].

## Supporting Information

Table S1 Relative telomere length association results from the GWAS and replication populations.
(XLSX)
